# Multisensory Systems Based on Perfluorosulfonic Acid Membranes Modified with Polyaniline and PEDOT for Multicomponent Analysis of Sulfacetamide Pharmaceuticals

**DOI:** 10.3390/polym14132545

**Published:** 2022-06-22

**Authors:** Anna Parshina, Anastasia Yelnikova, Tatyana Titova, Tatyana Kolganova, Polina Yurova, Irina Stenina, Olga Bobreshova, Andrey Yaroslavtsev

**Affiliations:** 1 Department of Analytical Chemistry, Voronezh State University, Voronezh 394018, Russia; anastasia_elnikova@outlook.com (A.Y.); tanyadenisova@list.ru (T.K.); bobreshova@chem.vsu.ru (O.B.); 2 Kurnakov Institute of General and Inorganic Chemistry RAS, Moscow 119991, Russia; titova_tatyana@mail.ru (T.T.); polina31415@mail.ru (P.Y.); irina_stenina@mail.ru (I.S.); yaroslav@igic.ras.ru (A.Y.)

**Keywords:** potentiometric multisensory system, cross-sensitivity, the Donnan potential, ionic transport, composite, perfluorosulfonic acid membrane, PEDOT, polyaniline, sulfacetamide, sulfanilamide

## Abstract

The degradation of sulfacetamide with the formation of sulfanilamide leads to a deterioration in the quality of pharmaceuticals. In this work, potentiometric sensors for the simultaneous determination of sulfanilamide, sulfacetamide and inorganic ions, and for assessing the degradation of pharmaceuticals were developed. A multisensory approach was used for this purpose. The sensor cross-sensitivity to related analytes was achieved using perfluorosulfonic acid membranes with poly(3,4-ethylenedioxythiophene) or polyaniline as dopants. The composite membranes were prepared by oxidative polymerization and characterized using FTIR and UV-Vis spectroscopy, and SEM. The influence of the preparation procedure and the dopant concentration on the membrane hydrophilicity, ion-exchange capacity, water uptake, and transport properties was investigated. The characteristics of the potentiometric sensors in aqueous solutions containing sulfanilamide, sulfacetamide and alkali metals ions in a wide pH range were established. The introduction of proton-acceptor groups and π-conjugated moieties into the perfluorosulfonic acid membranes increased the sensor sensitivity to organic analytes. The relative errors of sulfacetamide and sulfanilamide determination in the UV-degraded eye drops were 1.2 to 1.4 and 1.7 to 4%, respectively, at relative standard deviation of 6 to 9%.

## 1. Introduction

Drug degradation leads to a deterioration in the quality of pharmaceuticals and complicates the investigation of drug substances pharmacokinetics [1,2]. Besides, the study of drug degradation is important for ecological monitoring [3,4]. Thus, it is of great interest to develop effective technologies to determine antibiotic residues in different aquatic media. Pharmaceuticals based on the 4-aminobenzenesulfonic acid derivatives are widely used in the medicine and veterinary industries. Improper storage can lead to the active component degradation and a reduction in antibacterial activity of pharmaceuticals. One of their main degradation products is sulfanilamide. This dictates the need for its simultaneous determination with other members of this class. The techniques of HPLC liquid chromatography [5], thin layer chromatography [5], capillary electrochromatography [6], electrophoresis [7,8], and spectrophotometry [9] are used for the determination of 4-aminobenzenesulfonic acid derivatives. For the analysis of media with complex matrixes, including those containing several 4-aminobenzenesulfonic acid derivatives, hybrid methods combining extraction and chromatography with tandem mass-spectrometric [10,11,12] or UV-detectors [13], extraction and electrophoresis with UV-detection [14], extraction and UV-Visible spectrometry [15,16] are commonly used. They have some limitations for routine analysis [17]. Electrochemical sensors are attractive tools for a rapid reagent-free on-site analysis. Voltammetric sensors based on carbon nanomaterials (graphite, graphene, carbon nanotubes) modified with nanoparticles of metals [18], zinc and nickel oxides [19,20,21,22,23], and other materials [24,25,26] have been actively developed to determine water-soluble 4-aminobenzenesulfonic acid derivatives. These works do not discuss a possibility of achieving a high resolution of related analyte oxidation peaks. To develop potentiometric sensors for the determination of water-soluble 4-aminobenzenesulfonic acid derivatives, plasticized polyvinyl chloride membranes containing anion-exchangers based on manganese (III) tetraphenylporphyrin chloride [27], cyclodextrin [27], tetradodecylammonium bromide [28,29], bis(triphenylphosphoranilidene)ammonium [29], iron(II)-phthalocyanine [29], a complex of sodium sulfoquinoxaline with 2,3,5-triphenyltetrazolium chloride [30] were used. Nevertheless, they are not highly selective to individual members of sulfanilamide class in real media. For the sulfanilamide determination, ionophores based on molecularly imprinted polymers (MIPs) [31,32,33] are also developed. However, there are drawbacks of MIPs, such as the difficulty of the bound analyte extraction, the difference in the constants of the repeated interaction of analyte and reactive centers, and its non-specific binding to the matrix material [34]. As a result, a steady decline in interest to them is observed. Despite a large number of publications devoted to determination of 4-aminobenzenesulfonic acid derivatives, there are practically no methods for their determination in the presence of the primary member of the class (sulfanilamide) in the UV-degraded pharmaceuticals [35,36].

The lack of selective ionophores for the potentiometric determination of related analytes can be compensated using a multisensory approach, in which the responses of cross-sensitive sensors (sensitive to more than one component) are processed using multidimensional mathematical methods [37,38]. Materials for multisensory systems can be adopted from other applications. Such materials could be based on the Nafion-type perfluorosulfonic acid membranes, due to their self-organizing system of hydrophilic pores and channels that are of the same size as organic analyte ions. Moreover, it can be affected by modification or physical treatment. A high mechanical and chemical stability of perfluorosulfonic acid membranes is an important factor for routine analysis. It provides their low proneness to fouling, fast and effective regeneration [39]. The possibility of simultaneous determination of dicarboxylic amino acid homologues in a wide pH range using an array of cross-sensitive DP-sensors (DP is the Donnan potential) based on the Nafion membranes and their Russian analogue MF-4SC membrane was shown elsewhere [40]. The simultaneous sensitivity of DP-sensors to anions of aspartic and glutamic acids and potassium cations was achieved by the introduction of silica nanoparticles surface-modified with amino moieties in the membrane pores. Moreover, membranes were thermally treated at different levels of relative humidity. It allowed varying the concentration and nature of reactive centers in a membrane as well as its microstructure and conductivity that affect the sorption efficiency of analytes by ion-exchange and non-exchange mechanisms. The prospects of investigation of composites based on perfluorosulfonic acid membranes are associated with the possibility of varying potentiometric sensitivity to organic analytes due to nonspecific ion-ionic, ion-dipole, and hydrophobic interactions with membrane [40,41].

Poly(3,4-ethylenedioxythiophene) (PEDOT) and polyaniline (PANI) are of great interest as dopants for the Nafion-type membranes. The use of Nafion/PEDOT and Nafion/PANI composites in membrane-electrode assemblies of hydrogen-air fuel cells provides an improvement in their performance by increasing proton conductivity, decreasing dehydration at low relative humidity and reducing gas permeability of composites comparing to the pristine Nafion membranes [42,43,44,45]. The proton-acceptor properties of PANI contribute to an increase in monovalent permselectivity of perfluorosulfonic acid membranes that can be used for electrodialysis water treatment [46]. A combination of adhesive and conducting properties of PEDOT or PANI with the Nafion selectivity to cations allows the use of composites based on them for an improvement in the stability and selectivity of electrochemical sensors [47,48,49,50]. A simple synthesis, mechanical and thermal stability of PANI and PEDOT are important for the mentioned applications. It should be specified that the introduction of polymers with proton-acceptor groups in perfluorosulfonic acid membranes can negatively affect the membrane properties owing to binding of sulfonic acid groups and blocking membrane pores and channels [51]. Therefore, a study of the influence of the dopant concentration and its polymerization method is important in optimizing the properties of composites and devices based on them. In this work, a possibility for the use of composites based on Nafion-117 and MF-4SC membranes modified with PANI or PEDOT by different methods in potentiometric sensors for the simultaneous determination of sulfacetamide and sulfanilamide in pharmaceuticals was investigated.

The aim of this work was the development of multisensory systems with DP-sensors based on perfluorosulfonic acid membranes containing PANI or PEDOT for simultaneous determination of sulfacetamide, sulfanilamide and sodium ions in a wide pH range, and their application for the quality control of pharmaceuticals.

## 2. Materials and Methods

### 2.1. Materials and Reagents

Commercial Nafion-117 (Sigma-Aldrich, Saint-Louis, MO, USA) and MF-4SC (Plastpolymer, Saint-Petersburg, Russia) perfluorosulfonic acid membranes, an isopropyl alcohol solution of the MF-4SC perfluorosulfonic acid polymer in the lithium form (10 wt%, equivalent weight is 1100; Plastpolymer, Saint-Petersburg, Russia), aniline hydrochloride (>99%, Merck, Darmstadt, Germany), 3,4-ethylenedioxythiophene (98%, Sigma-Aldrich, Saint-Louis, MO, USA), ammonium persulfate (>98%, Sigma-Aldrich, Saint-Louis, MO, USA), hydrochloric acid (special purity grade, Chimmed, Moscow, Russia), potassium chloride (reagent grade, Chimmed, Moscow, Russia), sodium hydroxide (standard titer, REAGENT, Moscow, Russia), sulfanilamide (4-aminobenzenesulfonamide, >99%, Sigma-Aldrich, Saint-Louis, MO, USA), sulfacetamide (N-[(4-aminophenyl)sulfonyl]acetamide, >99%, Sigma-Aldrich, Saint-Louis, MO, USA), “Sulfacyl sodium-SOLOpharm” eye drops (Grotex, Saint-Petersburg, Russia), deionized water (resistance 18.2 MΩ, pH 5.41 ± 0.05) were used.

### 2.2. Preparation of Model Solutions

The characteristics of DP-sensors were established in solutions containing sulfanilamide (SA), sulfacetamide (SAA), and NaOH with various concentration ratios of the components ranging from 1.0 × 10^−4^ to 1.0 × 10^−2^ M. The solution composition included uncharged particles of sulfanilamide, anions of sulfacetamide, sodium cations, and water dissociation products (pH 4.76–10.70). A concentration matrix of calibration solutions was organized in a manner that the correlation coefficients between the negative decimal logarithm of the concentrations of sulfanilamide (pSA), sulfacetamide (pSAA) and sodium cations (pNa), as well as pH of the solutions were minimal. To prevent poor conditioning of the calibration equation, the correlation coefficients between the factors were not greater than 0.5, and the determinant of the correlation coefficient matrix was not less than 0.5. The values of the pairwise correlation coefficients (r_ij_) between the factors pSA, pSAA, pNa, pH, as well as the determinant of the correlation coefficient matrix (det r) are presented in Table 1.

The compositions of the model solutions were the same as those of the calibration solutions. The values of the DP-sensor responses for them were obtained independently (they were not included in the samples used in the calibration).

### 2.3. Pharmaceutical Pretreatment

“Sulfacyl sodium-SOLOpharm” eye drops contain sodium sulfacetamide monohydrate (the concentration of sodium sulfacetamide is 200 mg/mL), sodium thiosulphate pentahydrate (1.0 mg/mL), hydrochloric acid for pH adjustment and purified water. It corresponds to 0.847 M of sulfacetamide anions and 0.851 M of Na^+^ cations.

The pharmaceutical was diluted 500 times with deionized water, and subjected to forced degradation by UV treatment with a quartz-mercury table-top irradiator OKN-11 (Russia) for 10 min. The obtained solutions were used for potentiometric measurements with no further dilution. To perform spectrophotometric measurements, additional 50-fold dilution was required. Moreover, the spectrophotometric analysis of the pharmaceutical solutions diluted 25,000 times without UV treatment was performed.

According to the preparation composition, the concentrations of SAA^−^ and Na^+^ ions in the 500-fold diluted pharmaceutical solution without UV treatment were 1.69 × 10^−3^ and 1.70 × 10^−3^ M, respectively, and in the 25,000 times diluted pharmaceutical solution without UV treatment—8.47 × 10^−5^ and 8.51 × 10^−5^ M, respectively.

### 2.4. Membrane Preparation

**Figure 1:**
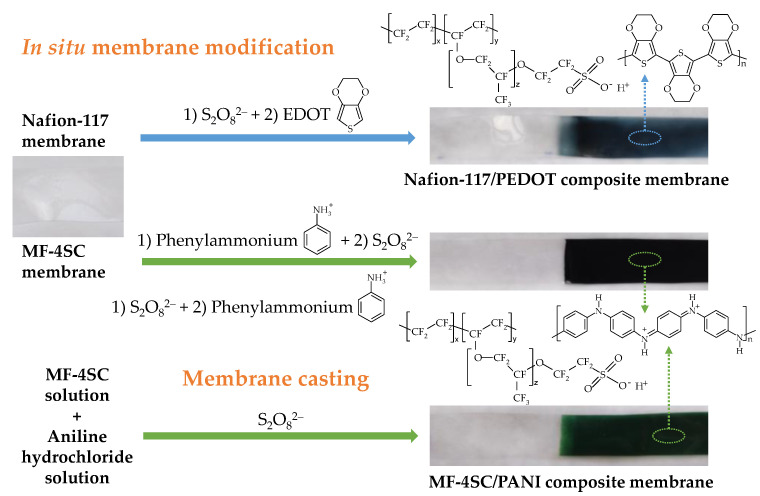
The scheme of the preparation of the Nafion-117/PEDOT and MF-4SC/PANI composite membranes with their photographic images.

Modified Nafion-117 and MF-4SC membranes with uniform (for equilibrium and transport property investigation) and gradient (for DP-sensors) distribution of PEDOT and PANI along the samples were manufactured. The scheme of the Nafion-117/PEDOT and MF-4SC/PANI composite membrane preparation is shown in Figure 1.

Commercial Nafion-117 and MF-4SC membranes were modified by in situ oxidative polymerization. For this purpose, they were treated with aqueous monomer and oxidant solutions at 25 °C. 3,4-ethylenedioxythiophene and aniline hydrochloride were monomers for PEDOT and PANI synthesis, respectively, and (NH_4_)_2_S_2_O_8_ was an oxidant in both cases. To prepare Nafion-117/PEDOT membrane samples by in situ method, Nafion-117 membranes were firstly treated with the oxidant solution for 2 h, washed with deionized water, and then treated with the monomer solution in 0.1 M hydrochloric acid for 2 h. The 3,4-ethylenedioxythiophene solution concentration was 0.002 or 0.01 M. The concentrations of (NH_4_)_2_S_2_O_8_ were 0.0025, 0.005, 0.0125, and 0.025 M. Thus, the ratio of the monomer and oxidant concentrations was 1/1.25 or 1/2.5. When manufacturing MF-4SC/PANI membranes by in situ method, the order of their treatment with the precursor solutions was varied. In the first case, MF-4SC membranes were treated with the monomer solution for 10 min and then with the oxidant solution for 10 h (Method N1). In the second case, MF-4SC membranes were sequentially treated with the solutions of the oxidant for 10 h, the monomer for 10 min and then the oxidant for 10 h (Method N2). The aniline hydrochloride concentrations were 0.005 and 0.01 M, and the ratio of the monomer and the oxidant concentrations was 1/1.25. Further, the membrane sample designations indicate the preparation method, monomer concentration and monomer/oxidant concentration ratio, e.g., Nafion-117/PEDOT (0.01 M, 1/1.25, in situ) or MF-4SC/PANI (0.01 M, 1/1.25, in situ, N1).

To prepare MF-4SC/PANI membranes by casting procedure, aqueous-alcoholic solutions of aniline hydrochloride and (NH_4_)_2_S_2_O_8_ were added to a solution of the MF-4SC perfluorosulfonic acid polymer at 25 °C under constant stirring. The monomer concentration in the solution was calculated to prepare membranes containing 0.5 and 1.0 wt% PANI, the water to isopropanol ratio was 1/2 (*v*/*v*). To complete the aniline polymerization, the mixture was stirred for 2 h. Then it was casted onto a glass surface and dried in air at 25 °C for 24 h, then at 50, 60, 70 °C for 2 h at each temperature for gradual solvent removal and a formation of a composite film. After removing from a glass surface, the films were hot-pressed under a pressure of 1.5 atm and at 110 °C for 5 min. Further, the membrane sample designations indicate the preparation method and PANI concentration, e.g., MF-4SC/PANI (1.0 wt%, casting).

The fabricated composite materials were bulk modified films with a thickness in a swollen state of 0.0130 ± 0.0003, 0.0124 ± 0.0003 and 0.0193 ± 0.0003 cm for membranes based on MF-4SC(casting), commercial MF-4SC and Nafion-117 membranes, respectively. Some photos of prepared composites are shown in Figure 1.

The pristine commercial Nafion-117 and MF-4SC membranes as well as unmodified MF-4SC membranes prepared by casting procedure from the MF-4SC solution were used as reference samples.

To prepare membranes with a dopant gradient distribution along the samples by in situ method, prepared films were immersed in the monomer and oxidant solutions only up to half their length. To cast gradient modified membranes, a lab-made glass cell was used. The perfluorosulfonic acid polymer solution was casted from one side, and the perfluorosulfonic acid polymer solution with the dopant—from another. Then membranes were formed the same way as uniform modified samples according to the methods described above. Thus, membranes of 6 cm in length with the dopant up to half of their length were prepared. At the same time, the boundary between the modified and unmodified parts was slightly blurred (the width of an intermediate part was no greater than 0.5 cm). The intermediate part does not introduce an error in the DP-sensor response, since the membrane ends were immersed in the test and reference solutions only to 0.3–0.5 cm. In this case, the unmodified end of a membrane was in contact with the reference solution. A dopant absence provided the composition closeness of the reference solution and the intrapore solution, levelling the potential difference at the corresponding interface.

To standardize conditions, the prepared membranes were conditioned according to the procedure described in [42,43]. The membrane samples were in the H^+^-form. To convert membranes to the K^+^-form, they were kept in 2 M KCl solution for 72 h, followed by washing in deionized water. The membranes were regenerated in the same way after long-term use (up to 3 months). After a series of repeated measurements (~100 times), the membranes were firstly kept in 0.1 M KCl solution for 30 min under constant stirring, then placed for storage in deionized water.

### 2.5. Apparatus and Experiment Procedure

Prepared composite membranes were characterized using UV-Visible spectroscopy, FTIR spectroscopy, and scanning electron microscopy (SEM).

UV-Visible-spectra of membranes were recorded using a Shimadzu UV-1800 spectrophotometer (Shimadzu, Kyoto, Japan) in the wavelength range from 190 to 1100 nm.

FTIR-spectra in attenuated total reflection (ATR) mode were recorded on a Nicolet iS5 FTIR spectrometer (Thermo Scientific, Waltham, MA, USA) using a diamond Specac Quest ATR add-on.

SEM images were obtained using a Tescan Amber instrument (Tescan, Brno, Czech Republic) equipped with an energy-dispersive X-ray spectroscopy (EDS) detector (Oxford Instruments, Bognor Regis, UK).

Static contact angle measurements were performed using a contact angle analyzer KRUSS DSA25 (KRUSS, Hamburg, Germany). Before measurement, the pristine Nafion-117 and MF-4SC membranes and the composite membranes were kept at 30% relative humidity for 4 days. A DI water drop of fixed volume (0.5 µL) was deposited onto the membrane surface. The images were taken by a digital high-resolution USB 3.0 camera. All measurements were conducted at least three times for each membrane and obtained data were averaged.

The equilibrium and transport properties of the membranes in the H^+^- and K^+^-form were investigated for the evaluation of the modification influence on their system of pores and channels.

The membrane water uptake was measured using a Netzsch-TG 209 F1 thermal balance (Netzsch, Selb, Germany) in the temperature range of 20–200 °C. The direct titration method was used for the ion-exchange capacity (IEC) determination. The IEC values were calculated per 1 g of a swollen membrane.

The conductivity measurements were performed using an Elins Z500 PRO impedance meter (Elins, Zelenograd, Russia) in the frequency range of 10^–2^ × 10^6^ Hz in potentiostatic mode with an amplitude of 80 mV in deionized water in the temperature range of 25–50 °C. The measurements were performed using graphite paper/membrane/graphite paper symmetric cells with an electrodes active surface area of ~1 cm^2^. The Nyquist plots were extrapolated to the active resistance axis to determine the membrane resistance.

The H^+^/K^+^ mutual diffusion and diffusion permeability were studied in a two-chamber cell separated by a membrane for 0.1 M KCl/0.1 M HCl and 0.1 M KCl/H_2_O solution systems, respectively. An Ekonix-Expert 001 pH-meter and Ekonix-Expert 002 conductometer (Ekonix-Expert, Moscow, Russia) were used to measure the pH solution and the concentration of KCl solution, respectively. The time of completing diffusion experiments was established when the pH or conductivity of the solution reached a stationary value.

Membranes in the K^+^-form were used in the DP-sensors. The cell scheme for the response evaluation of DP-sensor systems based on membranes with different compositions is thoroughly described elsewhere [40,41]). The distance between boundaries of an ion-exchange membrane with a reference and test solutions corresponds to the membrane length (6 cm) and not to its thickness as in traditional potentiometric sensors with inner filling. It allows to minimize ion transmembrane transfer, therefore the sensor response is the Donnan potential (DP) [52]. Membranes are not rigidly fixed into the sensor bodies. They connect reference and test solutions as bridges. Using a multi-section membrane cell, the modified end of the membrane was immersed in the central section with the test solution, and the other (unmodified) end of the membrane was immersed in the separate sections with the reference solutions. The ESr-10103 silver chloride electrodes and the ES-10301/4 glass electrode (Econix-Expert, Moscow, Russia) were used. Using a multichannel potentiometer, a potential difference was measured between a silver chloride electrode (connected to the reference electrode input) immersed in the test solution and silver chloride electrodes (connected to the measurement inputs) immersed in the reference solutions. Additionally, the pH of the test solution was measured simultaneously. Chronopotentiometry was used to evaluate the response time and stability of the DP-sensors. The voltages of several circuits (1) were measured:

Ag|AgCl, sat. KCl|1M KCl|membrane|test solution|sat. KCl, AgCl|Ag(1)

Spectrophotometric analysis of the pharmaceuticals was performed according to the method described elsewhere [35] using a Shimadzu UV-1800 spectrometer. Calibration for the analysis of the preparation without UV treatment was performed at λ = 269 nm in sulfacetamide solutions containing sodium-acetate buffer (pH = 4.0). A system of calibration equations for the analysis of the preparation treated with UV radiation was determined at λ = 258 nm and λ = 269 nm in solutions containing sulfanilamide, sulfacetamide, and sodium-acetate buffer (pH = 4.0). The wavelengths of 258 and 269 nm correspond to the absorbance maximums of the individual solutions of sulfanilamide and sulfacetamide, respectively.

### 2.6. Data Processing Procedure

For analysis objects with a relatively simple matrix and known qualitative composition, multivariate regression analysis can be successfully used as a calibration method for cross-sensitive sensors. To calibrate the DP-sensors in multicomponent solutions, regression analysis was used. The influence of all components of the solutions, capable of ion-ionic, ion-molecular and hydrophobic interactions, on the DP-sensor response was taken into account in the calibration Equation (2).

(2) $$\Delta \varphi_{D}=b_{0}+b_{1}\cdot pNa+b_{2}\cdot pH+b_{3}\cdot pSA+b_{4}\cdot pSAA,$$

where ∆*φ_D_*—the value of the DP-sensor response, mV; *pNa*—the negative decimal logarithm of the molar concentration of Na^+^; *pSA*—the negative decimal logarithm of the molar concentration of SA; *pSAA*—the negative decimal logarithm of the molar concentration of SAA^−^; *b_0_*—the constant term of the calibration equation, mV; *b_i_*—the sensitivity coefficient of the DP-sensor to the *i*-th component, mV/p*c*.

The coefficients of the calibration equations were calculated by the least-squares method performing matrix operations (number of calibration solutions *k* = 12):

(3) $$\left[\begin{array}{c} b_{0}\\ b_{1}\\ b_{3}\\ b_{4} \end{array}]={\left({\left[\begin{array}{c} 1;pNa_{1};pH_{1};pSA_{1};pSAA_{1}\\ 1;pNa_{2};pH_{2};pSA_{2};pSAA_{2}\\ \cdots \\ 1;pNa_{k};pH_{k};pSA_{k};pSAA_{k} \end{array}\right]}^{T}[\begin{array}{c} 1;pNa_{1};pH_{1};pSA_{1};pSAA_{1}\\ 1;pNa_{2};pH_{2};pSA_{2};pSAA_{2}\\ \cdots \\ 1;pNa_{k};pH_{k};pSA_{k};pSAA_{k} \end{array}]\right)}^{-1}{\left[\begin{array}{c} 1;pNa_{1};pH_{1};pSA_{1};pSAA_{1}\\ 1;pNa_{2};pH_{2};pSA_{2};pSAA_{2}\\ \cdots \\ 1;pNa_{k};pH_{k};pSA_{k};pSAA_{k} \end{array}\right]}^{T}[\begin{array}{c} \Delta \varphi_{D_{1}}\\ \Delta \varphi_{D_{2}}\\ \cdots \\ \Delta \varphi_{D_{k}} \end{array}\right]$$

The significance of the calibration equation coefficients was evaluated by Student’s *t*-test. To control the adequacy of the calibration equations, the variance of reproducibility and the variance of adequacy by Fisher’s F-criterion were compared. The scatter of the experimental values of the DP-sensor response relative to those predicted by the calibration equations was less than 2%.

The DP-sensors with the minimal correlation between their responses according to the r-criterion were chosen for multisensory systems (the number of sensors in an array was the same as the number of analytes).

Limits of detection (LOD) of analytes for a DP-sensor array were estimated by the “3σ” rule as minimal concentration, with which the value of sensor response in solution differs from the value of sensor response in deionized water more than 3 values of standard deviation of the response in the deionized water.

A system of three calibration equations of DP-sensor array was solved by a matrix approach to calculate the analyte concentrations in the analysis object (4). The responses of the DP-sensor array as well as the pH of the analysis object were experimental data for concentration determination.

(4) $$\left[\begin{array}{c} pNa\\ pSA\\ pSAA \end{array}\right]=\left[\begin{array}{c} b_{1}^{\left(1\right)}b_{3}^{\left(1\right)}b_{4}^{\left(1\right)}\\ b_{1}^{\left(2\right)}b_{3}^{\left(2\right)}b_{4}^{\left(2\right)}\\ b_{1}^{\left(3\right)}b_{3}^{\left(3\right)}b_{4}^{\left(3\right)} \end{array}\right]\cdot \left[\begin{array}{c} \Delta \varphi_{D}^{\left(1\right)}-b_{0}^{\left(1\right)}-b_{2}^{\left(1\right)}\cdot pH\\ \Delta \varphi_{D}^{\left(2\right)}-b_{0}^{\left(2\right)}-b_{2}^{\left(3\right)}\cdot pH\\ \Delta \varphi_{D}^{\left(3\right)}-b_{0}^{\left(2\right)}-b_{2}^{\left(3\right)}\cdot pH \end{array}\right],\begin{array}{c} c_{Na}=10^{-pNa},\;M\\ c_{SA}=10^{-pSA},\;M\\ c_{SAA}=10^{-pSAA},\;M \end{array}$$

where the upper index indicates the DP-sensor number in a multisensory system.

The calibration equations for spectrophotometric analysis were also obtained by regression analysis. To determine sulfacetamide in the pharmaceutical solutions without UV treatment, concentration dependence of the solution absorbance ranging from 1.0 × 10^−5^ to 5.0 × 10^−5^ M was obtained (5). For simultaneous determination of sulfanilamide and sulfacetamide in the forced degraded pharmaceutical solutions, a system of the calibration equations was obtained in solutions containing sulfanilamide from 0.2 × 10^−5^ to 0.8 × 10^−5^ M and sulfacetamide from 1.0 × 10^−5^ to 4.0 × 10^−5^ M (6). The ranges of the analyte concentrations corresponded with linear parts of the calibration dependences.

(5) $$A_{269}=16.8\cdot 10^{3}\cdot c_{SAA}.$$

(6) $$\{\begin{array}{c} A_{258}=12.5\cdot 10^{3}\cdot c_{SAA}+16.6\cdot 10^{3}\cdot c_{SA},\\ A_{269}=15.9\cdot 10^{3}\cdot c_{SAA}+14.8\cdot 10^{3}\cdot c_{SA}. \end{array}$$

where *A_n_*—the absorbance of the solution at *n* nm wavelength.

To evaluate the accuracy, the results of the potentiometric analysis of preparation were compared with its composition declared by the manufacturer and with the results of the spectrophotometric analysis.

## 3. Results and Discussion

### 3.1. Properties of Membranes

**Figure 2:**
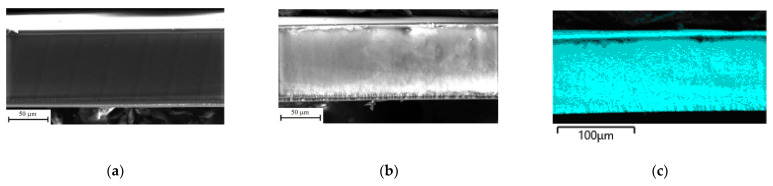

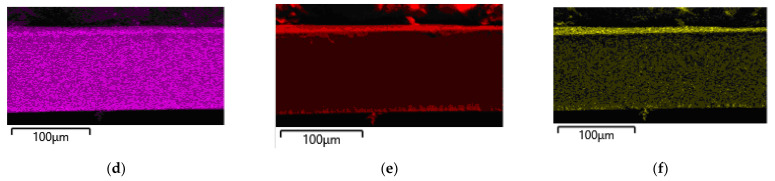
SEM images of cross-sections of the MF-4SC (**a**) and MF-4SC/PANI (0.005 M, 1/1.25, in situ, N2) (**b**) membranes; the EDX maps of F (**c**), S (**d**), C (**e**), N (**f**) for MF-4SC/PANI (0.005M, 1/1.25, in situ, N2) membrane.

The pristine and composite membranes are homogeneous with similar morphologies (Figure 2). There are no visible particles of PEDOT or PANI on the surface of the prepared composite membranes. The composition (set of elements) of perfluorosulfonic acid polymers (Nafion-117 and MF-4SC) and PEDOT or PANI are identical with the exception of fluorine in the case of PEDOT and fluorine and nitrogen in the case of PANI, and, thus, it is difficult to expect any differences on the SEM images.

EDX mapping of the cross-section of the MF-4SC/PANI (0.005M, 1/1.25, in situ, N2) membrane shows a uniform distribution of fluorine, carbon, sulfur and nitrogen over the membrane thickness indicating a uniform distribution of PANI in the composite membrane (Figure 2). It can be observed that there is more nitrogen in the membrane surface layers than in the membrane bulk due to the deposition of PANI in them.

**Figure 3:**
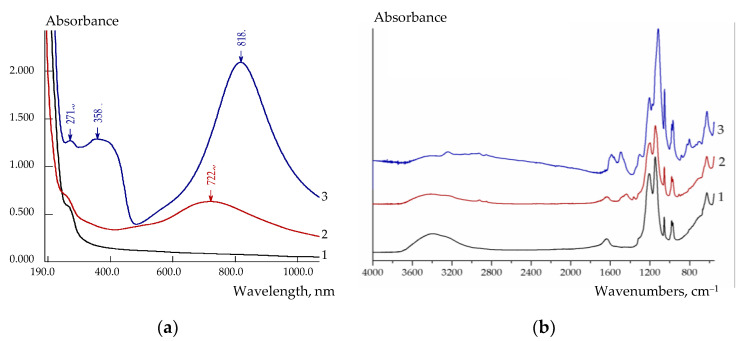
UV-Visible-spectra (**a**) and FTIR-spectra (**b**) of the Nafion-117 (pristine, commercial membrane) (1), Nafion-117/PEDOT (0.01M, 1/2.5, in situ) (2), MF-4SC/PANI (0.005 M, 1/1.25, in situ, N2) (3) composite membranes.

The presence of PEDOT or PANI in the membranes was evidenced not only by the uniform coloration of the membranes over the thickness (Figure 1), but also by UV-Vis- and FTIR-spectra of the composite membranes (Figure 3). Unlike the pristine membrane, there are peaks at 722 and 818 nm in the UV-Vis-spectra of Nafion-117/PEDOT and MF-4SC/PANI membranes, respectively. A peak batachromic shift, corresponding to the absorbance of blue-green and green colored substances, is typical for these polymers [53,54]. It is due to simultaneous presence of a conjugated system of double bonds and chromophoric groups with an unshared electron pair (=S, ≡N) in their structure. The characteristic peaks of benzene ring observed in MF-4SC/PANI membrane UV-Vis-spectrum are red-shifted (271 and 358 nm) because of the same reason (Figure 3a).

FTIR spectra of the Nafion-117/PEDOT composite membranes are similar to that of the pristine Nafion-117 membrane (Figure 3b) due to a low PEDOT content and the overlapping of a significant number of bands of poly(3,4-ethylenedioxythiophene) and perfluorosulfonic acid polymer [42,55]. However, in the regions of 900, 1400–1460 and 2900–3000 cm^−1^, new bands appeared assigned to the stretching vibrations of the C-S, C=C bonds of the thiophene ring and C-H bonds, respectively.

In the case of the in situ polymerization of aniline in the MF-4SC membrane, the intensity of the band at 3240 cm^−1^ assigned to the N-H stretching vibrations, as well as the band attributed to the C-H stretching vibrations (the region of 2800–3000 cm^−1^) increases (Figure 3b). Moreover, in FTIR spectra of the MF-4SC/PANI composites, there are other bands typical of polyaniline: at 1170 and 800 cm^−1^ (C–H in-plane and out-of-plane bending vibrations, respectively), 1490 and 1590 cm^−1^ (benzoic ring C-C (B-band) and quinoid ring C=C (Q-band) stretching mode, respectively) [56]. The intensities of the B- and Q-bands are close to each other (Figure 3b) indicating equivalent contents of amine and quinoneimine units and the formation of a high-conductive form of PANI (emeraldine). The intensity of the band at 1300 cm^−1^ associated with the C–N stretching vibration also increases. Near the peak at 1145 cm^−1^ (the asymmetric –SO_3_^−^ stretching vibration mode mixed with the symmetric C-F stretching vibration mode of perfluorosulfonic acid membrane) [55], the strongest peak of the whole spectrum appeared at 1118 cm^−1^, which can be attributed to the vibrational mode of Q=NH^+^-B structure of PANI [56].

The equilibrium and transport properties of composite materials depend on the dopant nature and the conditions of its formation in the membrane. As a result of in situ oxidative polymerization of 3,4-ethylenedioxythiophene in Nafion-117, composites with a low dopant content were prepared [42,57]. This is due to low sorption of both monomer and oxidant during the membrane modification since the oxidant (S_2_O_8_^2−^) is a co-ion for the Nafion cation-exchange membrane, and the solubility of 3,4-ethylenedioxythiophene even in HCl solution is low. The presence of PEDOT in the membranes decreases their IEC (from 0.65 mmol/g for the pristine membrane, to 0.59 mmol/g for the membrane with the highest PEDOT content). It can be due to the exclusion of some protons from ion exchange owing to the formation of hydrogen bonds between the membrane sulfonic acid groups and sulfur atoms of PEDOT. The water uptake of membranes changes insignificantly, and their diffusion permeability slightly increases. For example, the diffusion permeability of membranes prepared using 0.002 M monomer solution is 7–9% higher than that of Nafion-117 [57]. Considering the Donnan anion exclusion decreases with decreasing IEC, a more pronounced increase in diffusion permeability with PEDOT introduction can be expected. The relatively small difference in the diffusion permeability can be explained by the displacement of some electroneutral solution from membrane pores by the dopant [58]. The ionic conductivity of the composite membranes in the K^+^-form is also close to that of the pristine membrane and decreases with increasing both monomer concentration and the oxidant excess [57]. At the same time, the conductivity of membranes in the H^+^-form increases significantly with increasing PEDOT content in a membrane (from 0.028 S/cm for the pristine membrane to 0.051 S/cm for Nafion-117/PEDOT (0.01M, 1/2.5, in situ)) despite a decrease in ion-exchange capacity [57]. This confirms that the membrane modification by PEDOT is effective and is consistent with the model of limited elasticity of the membrane pore walls, according to which an increase in the volume of pores with the introduction of a dopant also leads to an expansion of the channels connecting them, which limit conductivity [59]. The possibility of PEDOT participation in proton transport due to the formed system of hydrogen bonds cannot be excluded.

**Figure 4:**
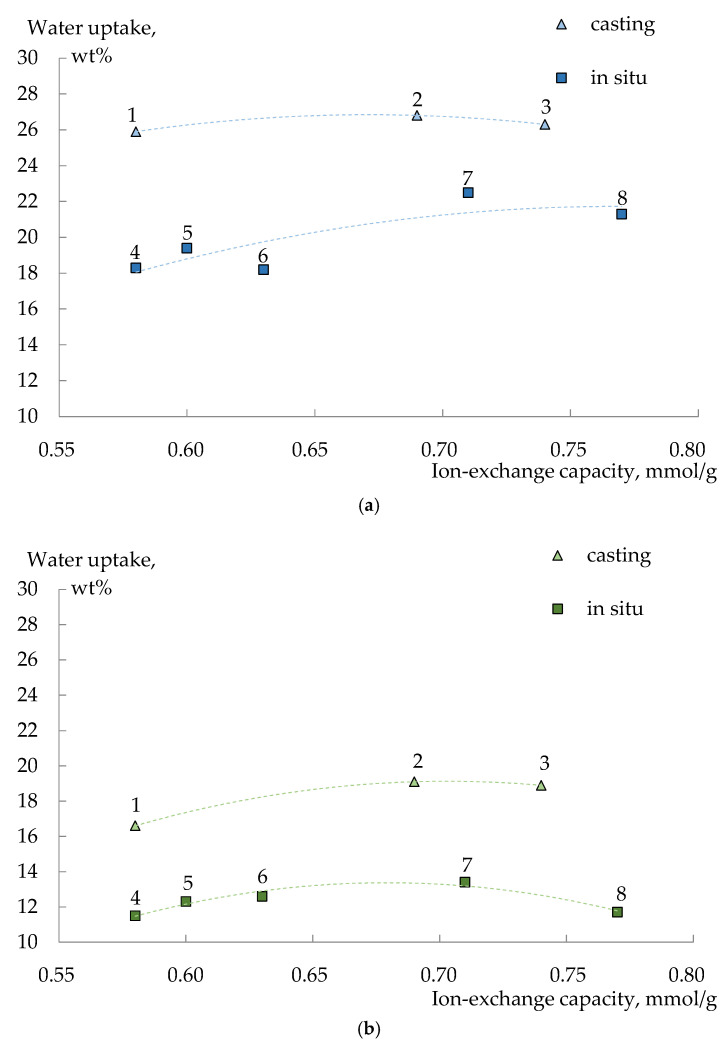
Ion-exchange capacity (±0.01 mmol/g) and water uptake (±0.5 wt%) of MF-4SC/PANI membranes in H^+^-form (**a**) and K^+^-form (**b**): 1—1.0 wt%, casting; 2—0.5 wt%, casting; 3—pristine, casting; 4—0.010 M, 1/1.25, in situ, N2; 5—0.005 M, 1/1.25, in situ, N2; 6—0.010 M, 1/1.25, in situ, N1; 7—0.005 M, 1/1.25, in situ, N1; 8—pristine commercial.

Modification of MF-4SC membranes with polyaniline provides a wider range of characteristics of the prepared composite membranes. It is due to the nature of monomer (aniline hydrochloride), which is a counter-ion to the membrane as well as different preparation methods (casting and in situ modification of a commercial membrane). The changes in IEC and water uptake of the membranes upon modification have common patterns for both preparation methods. IEC of the membranes decreases proportionally to the aniline hydrochloride concentration used for their preparation. Lower IEC values were obtained for the membranes prepared in situ method with the membrane treatment first by the oxidant solution and then with a monomer solution. The water uptake of the MF-4SC/PANI membranes increases when a small amount of PANI is introduced, and then it decreases to the values lower or close to those of the pristine membrane. Apparently, a small PANI amount enables membrane pore widening. At the same time, the increase in the dopant content provides their overlapping and cross-linking due to the formation of hydrogen bonds between sulfonic acid groups of the membrane and amino groups of PANI, which are more basic compared to sulfur atoms of PEDOT. This is also consistent with the model of limited elasticity of the membrane pore walls [59] and leads to a nonmonotonic dependence of water uptake on IEC (Figure 4).

**Figure 5:**
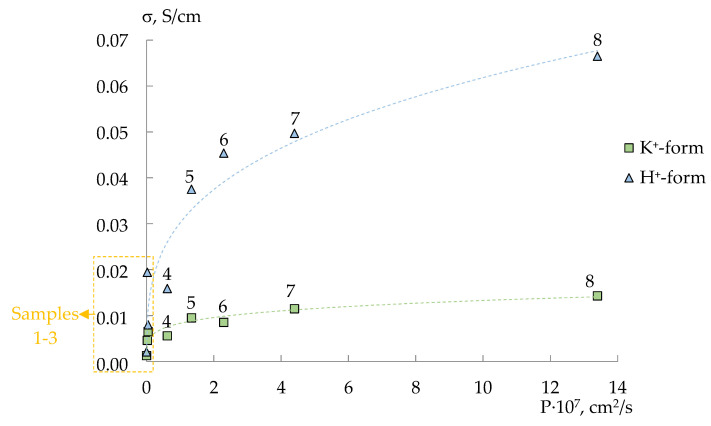
Conductivity (σ, S/cm) at 30 °C in deionized water and diffusion permeability (P 0.1 KCl/H_2_O, cm^2^/s) of MF-4SC/PANI membranes in the H^+^-form and K^+^-form: 1—0.010 M, 1/1.25, in situ, N1; 2—0.005 M, 1/1.25, in situ, N1; 3—0.010 M, 1/1.25, in situ, N2; 4—0.005 M, 1/1.25, in situ, N2; 5—pristine commercial; 6—pristine, casting; 7—0.5 wt%, casting; 8—1.0 wt%, casting. The relative errors in the estimation of conductivity and diffusion permeability are below 10 and 1.0%.

All MF-4SC/PANI membranes in the H^+^-form and K^+^-form are characterized by a symbate change in ionic conductivity and diffusion permeability. The ionic conductivity of membranes in the H^+^-form is 1.2–5 times higher than that of membranes in the K^+^-form and increases more significantly with an increase in the diffusion permeability. At the same time, the rate of ionic transport of the casted membranes is higher than that of the samples based on the commercial MF-4SC membrane and increases with increasing dopant content (Figure 5). Whereas the opposite dependence for the commercial MF-4SC membranes modified by in situ method is observed. Interestingly, this is more pronounced for the membranes first treated with the monomer solution and then with the oxidant solution (Figure 5).

A difference in morphology of perfluorosulfonic acid polymer in isopropyl alcohol solution and melt (commercial membranes prepared by extrusion) results in larger pore size and wider pore size distribution for cast membranes. For membranes casted from the MF-4SC solution with polyaniline, hydrophilic membrane clusters formed as a result of the self-organization contain not only sulfonic acid groups of perfluorosulfonic acid polymer but also PANI bound to them. It can lead to a larger size of pores and channels connecting them and a more uniform distribution of the dopant. This promotes higher water uptake and facilitates the transport of counter- and co-ions across the membrane (Figure 5) [59,60,61]. Besides, it provides the formation of more developed system of hydrogen bonds with participation of PANI and sulfonic acid groups of perfluorosulfonic acid polymer as evidenced by a sharp increase in conductivity of membranes in the H^+^-form.

The composite membrane formation by in situ method occurs differently. MF-4SC membranes sorb phenylammonium cations well and S_2_O_8_^2−^ anions much worse. It was established that the membrane saturation with phenylammonium cations was reached in a couple of minutes if the membrane separated aniline hydrochloride solution and deionized water. Therefore, when the membranes saturated with the monomer cations are treated with the oxidant solution (N1 series samples), polymerization of PANI begins in a thin surface layer of the membrane, where S_2_O_8_^2−^ anions can easily diffuse. It leads to PANI accumulation in the surface layer, and further aniline polymerization is blocked. The concentration of the oxidant anions in the membranes treated by (NH_4_)_2_S_2_O_8_ solution (N2 series samples) is low. Therefore, polymerization occurs only when a sufficient amount of oxidized molecules of aniline and radicals are formed [43]. This results in a more uniform distribution of PANI over the membrane sample. A more pronounced decrease in conductivity and diffusion permeability of the N1 series membranes indicates a higher PANI concentration in them (Figure 5). At the same time, IEC of these membranes decreases less than for the N2 series membranes. This may be due to the fact that, in these samples, surface sulfonic acid groups are predominantly blocked, while in the membrane bulk they remain available for ion exchange.

The assumptions made on the dopants distribution in a membrane and their influence on the system of pores and channels are in good agreement with the results of membrane hydrophilicity/hydrophobicity studies. The surface hydrophilicity/hydrophobicity of membranes was investigated by water contact angle measurements by the sessile drop method. The contact angle of the pristine commercial Nafion-117 and MF-4SC membranes is rather large (95.0 ± 0.3° and 92.3 ± 0.5°, respectively), because of hydrophobic C_X_F_Y_ chain and a small number of hydrophilic –SO_3_H groups in the perluorosulfonic acid polymers. However, the casted MF-4SC membrane is more hydrophilic showing a smaller contact angle (84.8 ± 0.5°).

Introduction of hydrophobic PEDOT in Nafion-117 results in a slight increase in contact angle of Nafion-117/PEDOT composite membranes (up to 100°). In the case of PANI introduction, the change in hybrophobicity is not so obvious. On the one hand, the N_X_H_Y_ fragments of polyaniline are hydrophilic, but they tend to form strong bridging bonds with –SO_3_H groups of perflurosolfonic acid polymer. The contact angle of casted MF-4SC/PANI membranes increases with increasing the PANI content: 98.4 ± 0.6° and 103.5 ± 0.5° for MF-4SC/PANI (0.5 wt%, casted) and MF-4SC/PANI (1 wt%, casted), respectively. This was attributed to a decrease in the amount of free hydrophilic—SO_3_H groups in these composite membranes due to ultimate contact between NH_2_ of aniline and -SO_3_H groups of perfluorosulfonic acid polymer formation and exclusion of a part of -SO_3_H groups from the membrane self-organization process at casting resulting in smaller hydrophilic clusters. In the case of in situ aniline polymerization in commercial MF-4SC/PANI membrane PANI forms in pre-formed hydrophilic membrane clusters and thus the prepared composite membranes show a smaller contact angle, which decreases with decreasing PANI content: 71.5 ± 0.5° for MF-4SC/PANI (0.01 M, 1/1.25, in situ, N1) and 65.8 ± 0.4° for MF-4SC/PANI (0.005 M, 1/1.25, in situ, N1).

### 3.2. Characteristics of the DP-Sensors

#### 3.2.1. Stability and Reproducibility of the DP-Sensors

The investigation of the response of the DP-sensors based on the pristine and composite membranes that were in contact with a solution containing 1.0 × 10^−3^ M of sulfanilamide, sulfacetamide, and NaOH for 1 h, shows that a couple of seconds is enough to reach its constant value. The further changes (the response drift) are comparable with the value scattering when repeating the experiment. The reproducibility variance values of the DP-sensors response are 10–27 mV^2^ in solutions containing sulfanilamide, sulfacetamide, and NaOH with concentrations from 1.0 × 10^−4^ to 1.0 × 10^−2^ M. The DP-sensors do not contain components capable of leaching from the membrane. However, the membrane fouling is possible due to the accumulation of organic analyte ions in the pores, affecting the sorption and transport properties of membranes [39]. To eliminate it in DP-sensors, membranes in the K^+^-form were used. The high affinity of perfluorosulfonic acid membranes to K^+^ ions provided fast and complete regeneration of the samples via equilibration with 0.1 M KCl solution. Moreover, the spatial separation of the membrane interfaces with the test solution and the reference solution of DP-sensors minimized the diffusion of components from the analyzed media into the membrane bulk. The re-estimation of the DP-sensor calibration characteristics showed no statistically significant difference after 1 year of the membrane use if the membrane was stored in deionized water and washed between measurement series.

#### 3.2.2. Cross-Sensitivity of the DP-Sensors

**Figure 6:**
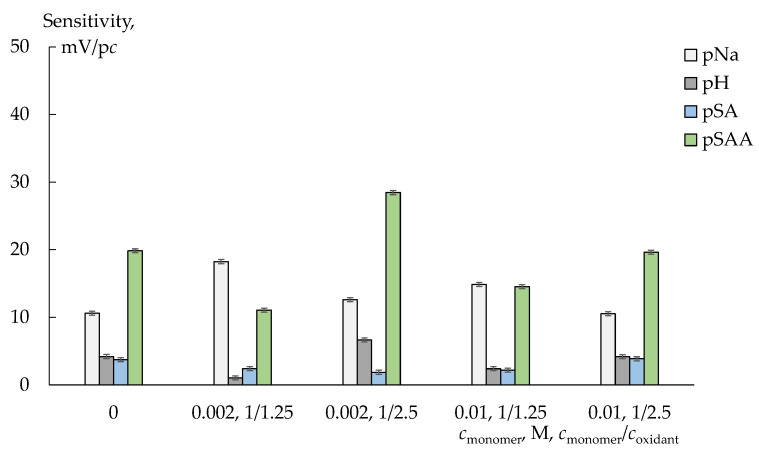
Sensitivity coefficients of DP-sensors based on Nafion-117/PEDOT(in situ, *c*_monomer_, M, *c*_monomer_*/c*_oxidant_) membranes to SAA^−^, SA, Na^+^ and water dissociation products in the concentration range 1.0 × 10^−4^–1.0 × 10^−2^ M and pH 4.76–10.70.

**Figure 7:**
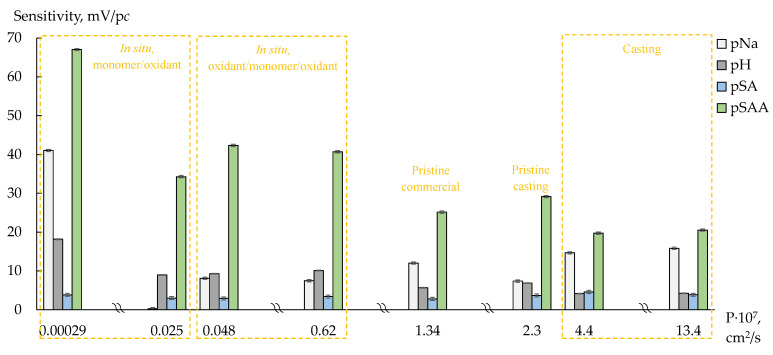
Sensitivity coefficients of DP-sensors based on MF-4SC/PANI membranes (preparation conditions are shown in the figure) to SAA^−^, SA, Na^+^ and water dissociation products in the concentration range 1.0 × 10^−4^–1.0 × 10^−2^ M and pH 4.76–10.70.

The DP-sensor sensitivity to sulfanilamide, which presented in solutions mainly in the uncharged form, is low, and it depends slightly on the membranes modification (Figure 6 and Figure 7). The DP-sensor sensitivity to sulfacetamide anions is higher and changes a lot depending on the dopant nature, concentration of monomer or oxidant and membrane preparation method (Figure 6 and Figure 7). A high sensitivity of the DP-sensors to the organic analytes in anion and zwitterion forms for the composites based on Nafion-type membranes has been described previously [40,41]. Since membranes are not absolutely selective, transport of organic anions and molecules into a cation-exchange membrane is possible. Anion sorption can be even more effective than that of uncharged molecules despite the Donnan exclusion. A facilitated transport of organic anions into the membrane is observed if other cations (Na^+^) than those of initial ionic membrane form (K^+^) are presented in the external solution [41]. In this case, anion transport into the membrane occurs via hydrogen bonds with hydration shells of cations involved in ion exchange. The states of the organic analytes, containing proton-donor and proton-acceptor groups in the structure, differ in the pore and external solutions because of the pH difference of these solutions due to the Donnan exclusion of OH^−^ ions. In the pristine membrane, they can participate in hydrogen bond formation with sulfonic acid groups of perfluorosulfonic acid polymer as well as hydration shells of counter-ions, which form the Debay layer. For the composite membranes containing PEDOT or PANI, electrostatic interactions and hydrogen bond formation between the analyte sulfonamide groups and proton-acceptor groups of the dopant as well as stacking interactions of the benzene ring of analytes and conjugated π-bonds of the dopants are possible.

The sensitivity of the DP-sensors based on Nafion-117/PEDOT membranes (prepared by in situ method) to sulfacetamide anions increases when increasing the concentration of the monomer solution used for their preparation (for the membranes prepared with the same monomer/oxidant ratio, Figure 6). An increase in the oxidant excess used for the membranes synthesis also leads to an increase in the DP-sensors sensitivity to sulfacetamide (if the membranes prepared with the same concentration of the monomer solution are compared, Figure 6). This is due to a decrease in IEC of the membranes and some facilitation of non-exchange sorption. Moreover, for the membranes prepared with different oxidant excess, the opposite correlation of the DP-sensors sensitivity to Na^+^ and sulfacetamide ions is observed. Taking into account slight changes in transport properties of these membranes, the most probable reason for it is a dopant distribution in the membrane, which can be more uniform due to the oxidant excess.

In the case of MF-4SC/PANI membranes prepared by different methods, there is a general trend of an increasing DP-sensor sensitivity to sulfacetamide anions with decreasing membranes diffusion permeability (Figure 7). At the same time, the sensitivity of DP-sensors to Na^+^ ions changes nonmonotonically (Figure 7). This effect can be explained taking into account the difference in the dopant (PANI) formation during the membrane synthesis. The highest sensitivity of DP-sensors to sulfacetamide anions is achieved for MF-4SC/PANI (in situ) membranes of N1 series prepared via saturation of the membranes with the monomer and then treated by the oxidant. An increased PANI concentration in the surface layers of the membrane apparently promotes the best conditions for the sorption of bulk organic anions. In this case, the concentration and availability of sorption centers for ion-ionic and stacking interactions with the analyte are maximal. The membrane casting from a homogeneous solution of perfluorosulfonic acid polymer and monomer (anyline hydrochloride) bound to sulfonic acid groups with further addition of oxidant results in the formation of a more uniform system of pores and channels with promoted non-selective transport as for inorganic co-ions. However, there is no increase in the DP-sensor sensitivity to sulfacetamide anions for these membranes. The reason can be in the steric hindrance of sorption of bulk organic anions due to pores cross-linking via hydrogen bonds between functional groups of PANI and perfluorosulfonic acid polymer. Moreover, the bonds between molecules of perfluorosulfonic acid polymer and PANI can reduce the elasticity of casted composite membranes.

Taking into account the cation-exchange nature of Nafion-117 and MF-4SC membranes, the DP-sensors sensitivity to Na^+^ cations was relatively low (Figure 6 and Figure 7). A decrease in the sensitivity of DP-sensors to Na^+^ cations and a simultaneous increase in the sensitivity to sulfacetamide anions are observed for unmodified membranes in a series: Nafion-117 (commercial), MF-4SC (commercial), MF-4SC (casting) membranes. In this series, a system of pores and channels becomes more developed. A decrease in the sensitivity of DP-sensors to Na^+^ cations is due to the same reasons as an increase in the sensitivity to organic anions when they are together in the test solutions. Coupled transfer of Na^+^ and sulfacetamide ions reduces the energy barrier for co-ion transition into membrane and increases it for counter-ion. At the same time, bulk ions of organic analyte in the membrane pores somehow inactivates membranes to cations due to steric factor and functional groups binding. This results in a decrease in the sensitivity of DP-sensors to Na^+^ cations with PANI introduction by in situ method. The MF-4SC/PANI (0.01M, 1/1.25, in situ, N1) membrane is an exception. It exhibits a high sensitivity of the DP-sensor to both Na^+^ cations and sulfacetamide anions. It should be noted that the diffusion permeability of this membrane is 2–3 times lower compared to other MF-4SC/PANI (in situ) membranes. Sulfanilamide interacts just with the surface located PANI, apparently due to low membrane permeability for anions, while sulfonic acid groups in the membrane bulk are available for ion-exchange.

The different distribution of sensitivity of DP-sensors to SAA^−^, SA, Na^+^ and water dissociation products provides the reduction in correlation between the responses of DP-sensors based on membranes with various composition and preparation method. It enables a possibility to organize DP-sensor arrays for simultaneous determination of analytes.

#### 3.2.3. Analysis of Model Solutions and Pharmaceuticals

Two systems of DP-sensors were chosen for the simultaneous determination of SAA^−^, SA and Na^+^ in the concentration range from 1.0 × 10^−4^ to 1.0 × 10^−2^ M in a wide range of pH. The first array of DP-sensors consisted of the MF-4SC/PANI (0.5 wt%, casting), MF-4SC/PANI (0.01M, 1/1.25, in situ, N1) and MF-4SC/PANI (0.01M, 1/1.25, in situ, N2) membranes. The second array was based on the MF-4SC/PANI (0.5 wt%, casting), MF-4SC/PANI (0.01M, 1/1.25, in situ, N1) and Nafion-117/PEDOT (0.002M, 1/1.25, in situ) membranes. Limits of detection of SAA^−^, SA and Na^+^ for the first array were 4.1 × 10^−6^, 1.0 × 10^−5^ and 3.0 × 10^−6^ M, respectively, for the second array—7.2 × 10^−6^, 1.0 × 10^−5^ and 5.4 × 10^−7^ M, respectively. Table 2 shows the systems of calibration equations, the scatter of experimental response values relative to those predicted by the calibration equations (ε, mV) and the variance of the response (D, mV^2^) for the chosen DP-sensor systems, as well as the characteristics of the model solutions with different ratio of the analyte concentrations in the pH range 4.76–10.70. The relative standard deviation (RSD) of the SAA^−^, SA and Na^+^ determination in the model solutions using the first array is 7–16%, the second array—5–20%. The relative errors of the SAA^−^, SA and Na^+^ determination using the first array are 1.7–4, 3–11 and 1.2–17%, the second array—0.5–21, 3–15 and 1.8–9%, respectively.

The multisensory systems were used for the analysis of the degraded “Sulfacyl sodium-SOLOpharm” preparation (eye drops). The concentrations of SAA^−^, SA and Na^+^ in the pharmaceutical solution diluted in 500-fold and treated with UV radiation determined with the use of the DP-sensor array based on MF-4SC/PANI (0.5 wt%, casting), MF-4SC/PANI (0.01 M, 1/1.25, in situ, N1), MF-4SC/PANI (0.01 M, 1/1.25, in situ, N2) membranes are (1.58 ± 0.15) × 10^−3^, (1.35 ± 0.17) × 10^−4^ and (1.75 ± 0.16) × 10^−3^ M at 6–8% RSD (Table 3). The concentrations of SAA^−^, SA and Na^+^ determined at the same conditions using the DP-sensor array based on MF-4SC/PANI (0.5 wt%, casting), MF-4SC/PANI (0.01 M, 1/1.25, in situ, N1), Nafion-117/PEDOT (0.002 M, 1/1.25, in situ) membranes are (1.57 ± 0.13) × 10^−3^, (1.33 ± 0.15) × 10^−4^ and (1.76 ± 0.15) × 10^−3^ M at 7–9% RSD (Table 3). This corresponds to the sulfacetamide sodium concentration of 185 ± 17 mg/mL and the sulfanilamide concentration of 11.6 ± 1.5 mg/mL in the preparation for the first DP-sensor array, and those of 185 ± 16 and 11.4 ± 1.3 mg/mL, respectively, for the second DP-sensor array.

The sodium sulfacetamide concentration decreases significantly via UV treatment (from 201 ± 2 to 185 ± 2 mg/mL, Table 3). At the same time, the sulfanilamide concentration in the pharmaceutical solution diluted in 500-fold, treated with UV radiation, and then diluted in 50-fold has the same value as an error of its determination.

The relative error of the sodium sulfacetamide determination in the preparation treated with UV radiation using the first and the second DP-sensor arrays relative to the concentration found by spectrophotometry is 1.4 and 1.2%, respectively. To evaluate the accuracy of the sulfanilamide determination, its concentration found by potentiometry was compared with the loss of the sulfacetamide concentration determined by spectrophotometry. The relative error of the sulfanilamide determination using the first and the second DP-sensor arrays is 1.7 and 4%, respectively. The relative error of the sodium ions determination relative to the content declared by the manufacturer is 0.7 and 4% for the first and the second DP-sensor arrays, respectively.

## 4. Conclusions

Multisensory systems for the simultaneous determination of sulfacetamide, sulfanilamide, and inorganic ions in the pharmaceuticals for the evaluation of a degradation degree of the active component were developed. The sensors cross-sensitivity to the related analytes was achieved using perfluorosulfonic acid membranes with PEDOT or PANI prepared by oxidative polymerization. The properties of composite membranes varied due to different precursor concentrations, the sequence of membrane treatment with precursor solutions and the methods of membrane preparation (casting procedure, in situ method). The obtained composite membranes were characterized using UV-Visible spectroscopy, FTIR spectroscopy, and SEM. Their hydrophilicity/hydrophobicity, as well as their equilibrium and transport properties in different ionic forms were investigated for the evaluation of the modification influence on the membrane system of pores and channels. The correlation between the diffusion permeability of the composite membranes and the sensor cross-sensitivity in solutions containing different forms of sulfanilamide and sulfacetamide and sodium cations in a wide pH range was established. The introduction of proton-acceptor groups and π-conjugated moieties in the membranes increased the sensitivity of DP-sensors to the organic analyte anions. This was more pronounced for membranes prepared via initial saturation with phenylammonium cations and further treated with the oxidant due to an increase in PANI concentration in the membrane surface layer. The application of composite membranes with different dopant content and prepared by different methods provided a low correlation between the responses of cross-sensitive sensors and the high accuracy of the analyte determination. The relative errors of sulfacetamide and sulfanilamide determination in the “Sulfacyl sodium-SOLOpharm” eye drops treated with UV radiation were 1.4 and 1.7%, while RSD was 6–8% for the first DP-sensor array, and 1.2 and 4% when RSD was 7–9% for the second DP-sensor array. The quality control of the pharmaceuticals was performed without probe pretreatment except for a little dilution. The possibility of long-term use of the DP-sensor arrays without re-calibration was shown.

## Figures and Tables

**Table 1:** The correlation coefficients (r_ij_) between the pairs of the factors: pSA (factor 1), pSAA (factor 2), pNa (factor 3), pH (factor 4) and the determinant of the correlation coefficient matrix (det r) (number of calibration solutions k = 12).

r_12_	r_13_	r_14_	r_23_	r_24_	r_34_	det r
0.1	0.3	0.2	0.4	0.5	0.5	0.5

**Table 2:** The characteristics of DP-sensor calibration equations based on MF-4SC/PANI (0.5 wt%, casting), MF-4SC/PANI (0.01 M, 1/1.25, in situ, N1), MF-4SC/PANI (0.01 M, 1/1.25, in situ, N2) membranes (array 1) and MF-4SC/PANI (0.5 wt%, casting), MF-4SC/PANI (0.01 M, 1/1.25, in situ, N1), Nafion-117/PEDOT (0.002 M, 1/1.25, in situ) membranes (array 2) as well as the characteristics of the SAA^−^, SA and Na^+^ determination in the model solutions (c = 1.0 × 10^−4^–1.0 × 10^−2^ M, pH 4.76–10.70).

**Array**	Calibration Equations Systems	ε, mV	D, mV^2^	Relative Error, %			RSD,% (*n* = 4, *p* = 0.95)		
				SAA^−^	SA	Na^+^	SAA^−^	SA	Na^+^
1	$\Delta \varphi_{D}^{\left(1\right)}=-65-14.7\cdot pNa+4.15\cdot pH-4.6\cdot pSA-19.7\cdot pSAA$	3	20	1.7–4	3–11	1.2–17	8–16	8–10	7–16
	$\Delta \varphi_{D}^{\left(2\right)}=-206.0+41.05\cdot pNa+18.16\cdot pH-3.84\cdot pSA-67.07\cdot pSAA$	4	10						
	$\Delta \varphi_{D}^{\left(2\right)}=-206.0+41.05\cdot pNa+18.16\cdot pH-3.84\cdot pSA-67.07\cdot pSAA$	3	12						
2	$\Delta \varphi_{D}^{\left(1\right)}=-65-14.7\cdot pNa+4.15\cdot pH-4.6\cdot pSA-19.7\cdot pSAA$	3	20	0.5–21	3–15	1.8–9	10–20	5–16	7–20
	$\Delta \varphi_{D}^{\left(2\right)}=-206.0+41.05\cdot pNa+18.16\cdot pH-3.84\cdot pSA-67.07\cdot pSAA$	4	10						
	$\Delta \varphi_{D}^{\left(3\right)}=-70-18.2\cdot pNa+1.03\cdot pH-2.4\cdot pSA-11.1\cdot pSAA$	4	27						

**Table 3:** The analysis of the “Sulfacyl sodium-SOLOpharm” eye drops (Grotex, Russia) using the DP-sensor systems based on MF-4SC/PANI (0.5 wt%, casting), MF-4SC/PANI (0.01 M, 1/1.25, in situ, N1), MF-4SC/PANI (0.01 M, 1/1.25, in situ, N2) membranes (array 1) and MF-4SC/PANI (0.5 wt%, casting), MF-4SC/PANI (0.01 M, 1/1.25, in situ, N1), Nafion-117/PEDOT (0.002 M, 1/1.25, in situ) membranes (array 2).

Method	Spectrophotometry		The DP-Sensor Array 1	The DP-Sensor Array 2
Pharmaceutical pretreatment	Dilution 1/25,000	UV treatment, dilution 1/25,000	UV treatment, dilution 1/500	UV treatment, dilution 1/500
c(SAA^−^), M (pharmaceutical solution)	(3.40 ± 0.02) × 10^−5^	(3.11 ± 0.04) × 10^−5^	(1.58 ± 0.15) × 10^−3^	(1.57 ± 0.13) × 10^−3^
c(SA), M (pharmaceutical solution)	-	insignificant	(1.35 ± 0.17) × 10^−4^	(1.33 ± 0.15) × 10^−4^
c(Na^+^), M (pharmaceutical solution)	-	-	(1.75 ± 0.16) × 10^−3^	(1.76 ± 0.15) × 10^−3^
RSD(SAA^−^), % (*n* = 4, *p* = 0.95)	0.3	1.5	6	7
RSD(SA), % (*n* = 4, *p* = 0.95)	-	-	8	9
RSD(Na^+^), % (*n* = 4, *p* = 0.95)	-	-	6	7
c(SAANa), mg/mL (preparation)	201 ± 2	185 ± 2	185 ± 17	185 ± 16
c(SA), mg/mL (preparation)	-	-	11.6 ± 1.5	11.4 ± 1.3
Relative error(SAANa), %	-	-	1.4	1.2
Relative error(SA), %	-	-	1.7	4

## Data Availability

Not applicable.
